# Supplementary material for: Multilevel Regulation of Peroxisomal Proteome by Post-Translational Modifications
Source: Int J Mol Sci. 2019 Oct 1;20(19):4881. doi: 10.3390/ijms20194881 (PMC6801620; doi:10.3390/ijms20194881)
Supplement: Supplementary file 1 [file ijms-20-04881-s001.pdf]

Supplemental Table S1: Putative peroxisomal protein targets of different PTMs

Locus	Description	PTM_Types	Number_PTM_Types
**PEROXISOME BIOGENESIS**			
AT1G01820	peroxin 11c	ac; ph	2
AT1G47750	peroxin 11a	ph	1
AT2G45740	peroxin 11d	na; nt; ph	3
AT3G21865	peroxin 22	na; nt; ph	3
AT3G07560	peroxin 13	ph	1
AT1G48635	peroxin 3	ph	1
AT2G26350	peroxin 10	ph	1
AT3G03490	peroxin 19-1	no; ph	2
AT5G17550	peroxin 19-2	na; nt	2
AT5G56290	peroxin 5	ph	1
AT5G62810	peroxin 14	na; nt; og; ph	4
AT4G33650	dynamin-related protein 3A	ac; nt; ph	3
AT1G06460	alpha-crystallin domain 32.1	ph	1
**AMINO ACID METABOLISM**			
AT5G16290	VALINE-TOLERANT 1	na; nt; ro	3
AT1G07180	alternative NAD(P)H dehydrogenase 1	ph; ub	2
AT1G11840	glyoxalase/bleomycin resistance protein/dioxygenase superfamily protein	ac; na; nt; sf	4
AT1G13440	glyceraldehyde-3-phosphate dehydrogenase C2	ac; mo; na; nt; ph	5
AT1G49350	pfkB-like carbohydrate kinase family protein	ph	1
AT1G49670	ARP protein	ac; na; no; ph; ro	5
AT1G52400	beta glucosidase 18	ac; ng; no	3
AT1G69960	serine/threonine protein phosphatase 2A	no; nt; ps	3
AT1G74910	ADP-glucose pyrophosphorylase family protein	na; ph	2
AT2G06050	oxophytodienoate-reductase 3	na; nt; ps	2
AT2G18630	transmembrane protein; putative (DUF677)	ph; ntyr	2
AT2G21660	cold; circadian rhythm; and rna binding 2	ac; na; nt; og; ph; sm; ub; ntyr	8

Suppl. Table S1 (cont.)

Locus	Description	PTM_Types	Number_PTM_Types
AT1G20480	AMP-dependent synthetase and ligase family protein	nt	1
AT1G20510	OPC-8:0 CoA ligase1	no	1
AT1G31910	GHMP kinase family protein	na; nt; ph; ps	4
AT1G65880	benzoyloxyglucosinolate 1	nt	1
AT2G26250	3-ketoacyl-CoA synthase 10	ng; nt; ph	3
AT2G29340	NAD-dependent epimerase/dehydratase family protein	no	1
AT2G30490	cinnamate-4-hydroxylase	ac	1
AT2G31790	UDP-Glycosyltransferase superfamily protein	ac	1
AT2G44490	Glycosyl hydrolase superfamily protein	ac; na; no; nt; ps	5
AT3G02780	isopentenyl pyrophosphate:dimethylallyl pyrophosphate isomerase 2	na; no; nt	3
AT3G14210	GDSL-like lipase/acylhydrolase superfamily protein	ac; mo; ng; nt	4
AT3G48170	aldehyde dehydrogenase 10A9	ac; mo; no; ph; ub	5
AT4G05160	AMP-dependent synthetase and ligase family protein	ac; na; nt; ps	4
AT4G16330	2-oxoglutarate (2OG) and Fe(II)-dependent oxygenase superfamily protein	no	1
AT5G16440	isopentenyl diphosphate isomerase 1	mo; na; nt	3
AT5G25980	glucoside glucohydrolase 2	ac; mo; ng; no; nt	5
AT5G26000	thioglucoside glucohydrolase 1	ac; mo; ng; no; nt	5
AT5G47720	Thiolase family protein	ac; nt	2
AT5G48230	acetoacetyl-CoA thiolase 2	ac; na; nt; ph; ro	5
**OTHERS**			
AT1G10430	protein phosphatase 2A-2	no; nt; ps	3
AT1G26930	Galactose oxidase/kelch repeat superfamily protein	ub	1
AT1G28320	protease-like protein	na	1
AT1G50510	indigoidine synthase A family protein	na; nt; ro; ub	4
AT1G52410	TSK-associating protein 1	ac; ng; nt	3
AT2G02510	NADH dehydrogenase (ubiquinone)s	na; nt; ub	3
AT2G14120	dynamin related protein	ac; na; ph	3
AT2G33830	Dormancy/auxin associated family	Ph	1
AT2G24580	FAD-dependent oxidoreductase family protein	no	1
AT1G76180	Dehydrin family protein (as chaperone)	ph	1

Suppl. Table S1 (cont.)

Locus	Description	PTM_Types	Number_PTM_Types
AT5G43940	S-nitrosoglutathione reductase	no; ph	2
AT2G26230	uricase / urate oxidase / nodulin 35	na; nt; ps	3
AT2G26300	G protein alpha subunit 1	ph	1
AT2G26340	hypothetical protein	ph	1
AT2G26540	uroporphyrinogen-III synthase family protein	ac; na; nt; ph	4
AT2G26560	phospholipase A 2A	na; no; nt	3
AT2G26730	Leucine-rich repeat protein kinase family protein	ng; ph	2
AT2G26750	alpha/beta-Hydrolases superfamily protein	ph	1
AT2G26890	DNAJ heat shock N-terminal domain-containing protein	ph; ro; ub	3
AT2G26910	pleiotropic drug resistance 4	ac; ng; ph	3
AT2G26980	CBL-interacting protein kinase 3	ph	1
AT2G27020	20S proteasome alpha subunit G1	ac; mo; na; nt; ph	5
AT2G27030	calmodulin 5	ac; na; no; nt; ph; ub	6
AT2G27130	Bifunctional inhibitor/lipid-transfer protein/seed storage 2S albumin superfamily protein	ro	1
AT2G27210	BRI1 suppressor 1 (BSU1)-like 3	no; ph; ro	3
AT2G27490	dephospho-CoA kinase family	ub	1
AT2G27600	AAA-type ATPase family protein	na; no; ub	3
AT2G27680	NAD(P)-linked oxidoreductase superfamily protein	ac; no	2
AT2G27730	copper ion binding protein	ac; nt; ub	3
AT2G27960	cyclin-dependent kinase-subunit 1	nt; ub	2
AT2G28000	chaperonin-60alpha	ac; mo; na; nt; ph; ub; ntyr	7
AT2G28680	RmlC-like cupins superfamily protein	na; ph	2
AT2G29330	tropinone reductase	no	1
AT2G29350	senescence-associated gene 13	na; no; nt	3
AT2G29370	NAD(P)-binding Rossmann-fold superfamily protein	ph	1
AT2G29450	glutathione S-transferase tau 5	ac; na; nt	3
AT2G29530	Tim10/DDP family zinc finger protein	na; nt; ro; ub	4
AT2G30520	Phototropic-responsive NPH3 family protein	ac; mo; na; nt; ph	5
AT2G30740	Protein kinase superfamily protein	ac; ph	2
AT2G30860	glutathione S-transferase PHI 9	ac; mo; na; nt; ph	5
AT2G30870	glutathione S-transferase PHI 10	ac; nt; ph	3

Suppl. Table S1 (cont.)

Locus	Description	PTM_Types	Number_PTM_Types
AT2G30930	hypothetical protein	ac; na; nt; ph	4
AT2G30950	FtsH extracellular protease family	ac; nt; ph	3
AT2G30970	aspartate aminotransferase 1	ac; no; nt	3
AT2G31670	Stress responsive alpha-beta barrel domain protein	ac; na; nt; ph; ps	5
AT2G31680	RAB GTPase homolog A5D	na; ph; ub	3
AT2G31725	FAM136A-like protein (DUF842)	na	1
AT2G31750	UDP-glucosyl transferase 74D1	no	1
AT2G31810	ACT domain-containing small subunit of acetolactate synthase protein	nt	1
AT2G31880	Leucine-rich repeat protein kinase family protein	ng; ph	2
AT2G32080	purin-rich alpha 1	na; og; ph	3
AT2G41790	Insulinase (Peptidase family M16) family protein	ac; na; ng; nt; ph; ps	6
AT2G42490	Copper amine oxidase family protein	na; nt	2
AT2G43940	S-adenosyl-L-methionine-dependent methyltransferases superfamily protein	na; ph	2
AT2G45690	shrunken seed protein (SSE1)	ph; ub	2
AT3G01910	sulfite oxidase	nt; ub; ps	3
AT3G02360	6-phosphogluconate dehydrogenase family protein	ac; no; nt; ph; ro; ps	6
AT3G09260	Glycosyl hydrolase superfamily protein	ac; ng; nt	3
AT3G12800	short-chain dehydrogenase-reductase B	na; nt; ps	3
AT3G15290	3-hydroxyacyl-CoA dehydrogenase family protein	ac; na; nt; ph	4
AT3G15950	DNA topoisomerase-like protein	ac; ng; nt	3
AT3G17420	glyoxysomal protein kinase 1	ph	1
AT3G19570	SNOWY COTYLEDON protein (DUF566)	ph	1
AT3G25800	protein phosphatase 2A subunit A2	ac; na; nt; ro; ps	5
AT3G46060	RAB GTPase homolog 8A	ac; ph	2
AT3G48140	B12D protein	ac	1
AT3G55040	glutathione transferase lambda 2	nt	1
AT3G55270	mitogen-activated protein kinase phosphatase 1	ph	1
AT3G56460	GroES-like zinc-binding alcohol dehydrogenase family protein	na; ph	2
AT3G56490	HIS triad family protein 3	no; ph; ub; ps	4
AT3G57090	Tetratricopeptide repeat (TPR)-like superfamily protein	ac; na; ph	3

Suppl. Table S1 (cont.)

Locus	Description	PTM_Types	Number_PTM_Types
AT3G57810	Cysteine proteinases superfamily protein	nt; ph; ub	3
AT3G58510	DEA(D/H)-box RNA helicase family protein	ac; na; no; nt; ph; ro; sf	7
AT3G58840	Tropomyosin-like protein	nt; ub	2
AT3G60680	DUF641 family protein (DUF641)	ph	1
AT3G61200	Thioesterase superfamily protein	na	1
AT3G62750	beta glucosidase 8	ng	1
AT4G00860	monopolar spindle protein (DUF1138)	ac; na; nt; ub	4
AT4G02340	alpha/beta-Hydrolases superfamily protein	no; sf	2
AT4G04470	Peroxisomal membrane 22 kDa (Mpv17/PMP22) family protein	na; nt	2
AT4G05020	NAD(P)H dehydrogenase B2	nt	1
AT4G05530	indole-3-butyric acid response 1	na; nt; ub; ps	4
AT4G09320	nucleoside diphosphate kinase	ac; na; no; nt; ph; ro; ub; ps; ca	8
AT4G12735	hypothetical protein	ph	1
AT4G12910	serine carboxypeptidase-like 20	ng; nt	2
AT4G14880	O-acetylserine (thiol) lyase (OAS-TL) isoform A1	ac; mo; no; nt; ph; ro; ub; ntyr	8
AT4G16566	histidine triad nucleotide-binding 4	na; nt	2
AT4G17520	Hyaluronan / mRNA binding family	ac; mo; nt; ph; ub	5
AT4G24160	alpha/beta-Hydrolases superfamily protein	na	1
AT4G30760	Putative endonuclease or glycosyl hydrolase	ph	1
AT4G36100	Sec1/munc18-like (SM) proteins superfamily	ph	1
AT4G36880	cysteine proteinase1	nt	1
AT4G39260	cold; circadian rhythm; and RNA binding 1	ac; mo; na; no; nt; ph; sm; ub; ca	9
AT4G39850	peroxisomal ABC transporter 1	nt; ph	2
AT5G03530	RAB GTPase homolog C2A	ph; ps	2
AT5G04040	Patatin-like phospholipase family protein	ph	1
AT5G04870	calcium dependent protein kinase 1	my; ph; ub	3
AT5G11520	aspartate aminotransferase 3	nt; ro; ub	3
AT5G11910	alpha/beta-Hydrolases superfamily protein	ph	1
AT5G12390	Tetratricopeptide repeat (TPR)-like superfamily protein	na	1
AT5G17380	Thiamine pyrophosphate dependent pyruvate decarboxylase family protein	ac; na; no; nt	4
AT5G17920	Cobalamin-independent synthase family protein	ac; mo; na; no; nt; ph; ub; ntyr	8

Suppl. Table S1 (cont.)

Locus	Description	PTM_Types	Number_PTM_Types
AT5G18910	Protein kinase superfamily protein	ph	1
AT5G20070	nudix hydrolase homolog 19	nt; ph; ro	3
AT5G23395	Cox19-like CHCH family protein	my; ro	2
AT5G24400	NagB/RpiA/CoA transferase-like superfamily protein	ac; na; nt; ph; ps	5
AT5G27520	peroxisomal adenine nucleotide carrier 2	ac; nt	2
AT5G35790	glucose-6-phosphate dehydrogenase 1	ac; nt; ps	3
AT5G41210	glutathione S-transferase THETA 1	ac; na; nt; ph; ps	5
AT5G41220	glutathione S-transferase THETA 3	ph	1
AT5G41240	glutathione S-transferase THETA 2	ph; ps	2
AT5G43280	delta(3;5);delta(2;4)-dienoyl-CoA isomerase 1	nt; ph	2
AT5G43940	GroES-like zinc-binding dehydrogenase family protein	ac; na; nt; ro; ub	5
AT5G47040	lon protease 2	na; nt; ph	3
AT5G47720	Thiolase family protein	ac; nt	2
AT5G48370	Thioesterase/thiol ester dehydrase-isomerase superfamily protein	ph	1
AT5G48545	histidine triad nucleotide-binding 3	ph	1
AT5G48880	peroxisomal 3-keto-acyl-CoA thiolase 2	ac; nt; ph; ps	4
AT5G53570	Ypt/Rab-GAP domain of gyp1p superfamily protein	ph	1
AT4G16340	(SPIKE1); GTP binding / GTPase binding / binding / guanyl-nucleotide exchange factor	ph	1
AT5G58220	transthyretin-like protein	na; nt; ph; ps	4
AT5G65940	beta-hydroxyisobutyryl-CoA hydrolase 1	na; nt; ph; ps	4
AT1G60140	Trehalose phosphate synthase	ph	1
AT2G29300	NAD(P)-binding Rossmann-fold superfamily protein	no	1
AT1G63900	E3 Ubiquitin ligase family protein	ph	1
AT2G30650	ATP-dependent caseinolytic (Clp) protease/crotonase family protein	ph	1
AT4G19010	AMP-dependent synthetase and ligase family protein	ph	1
AT5G63380	AMP-dependent synthetase and ligase family protein	ph	1

Carbonylation (ca), Lysine Acetylation (ac),Lysine Ubiquitination (ub), Methionine Oxidation (mo), N-glycosylation (ng), N-terminal Acetylation (na), N-terminus Proteolysis (nt), N-terminal Ubiquitination (nu), O-GlcNAcylation (og), Phosphorylation (ph), Reversible Cysteine Oxidation (ro), S-Glutathionylation (sg), S-Nitrosylation (no), persulfidation (ps), sulfenilation (sf), nitration (ntyr)
